# Enlarged vestibular aqueduct and Mondini Malformation: audiological, clinical, radiologic and genetic features

**DOI:** 10.1007/s00405-020-06333-9

**Published:** 2020-09-10

**Authors:** F. Forli, F. Lazzerini, G. Auletta, L. Bruschini, S. Berrettini

**Affiliations:** 1 Otolaryngology, Audiology and Phoniatrics Unit, University of Pisa, Pisa, Italy; 2 Department of Clinical Science, Intervention and Technology, Karolinska Institutet, Stockholm, Sweden; 3 UOC Audiologia, DAI Testa Collo, AOU Federico II, Naples, Italy

**Keywords:** Enlarged vestibular aqueduct, Mondini Malformation, Hearing loss, Inner ear malformation, Pendred Syndrome

## Abstract

**Purpose:**

When referring to enlarged vestibular aqueduct (EVA) we should differentiate between nonsyndromic enlarged vestibular aqueduct (NSEVA) and Pendred Syndrome (PDS), a disease continuum associated with pathogenic sequence variants of Pendrin’s Gene (SLC26A4) in about half of the cases. The study was aimed to analyse the clinical and audiological features of a monocentric cohort of Caucasian patients with NSEVA/PDS, their genetic assessment and morphological inner ear features.

**Methods:**

We retrospectively reviewed the audiologic, genetic and anamnestic data of 66 patients with NSEVA/PDS followed by our audiology service.

**Results:**

SLC26A4 mutations was significantly correlated with the presence of PDS rather than NSEVA (*p* < 0.019), with the expression of inner ear malformations (*p* < 0.001) and with different severity of hearing loss (*p* = 0.001). Furthermore, patients with PDS showed significantly worse pure tone audiometry (PTA) than patients with NSEVA (*p* = 0.001). Anatomically normal ears presented significantly better PTA than ears associated with Mondini Malformation or isolated EVA (*p* < 0.001), but no statistically significative differences have been observed in PTA between patients with Mondini Malformation and isolated EVA.

**Conclusion:**

NSEVA/PDS must be investigated in all the congenital hearing loss, but also in progressive, late onset, stepwise forms. Even mixed or fluctuating hearing loss may constitute a sign of a NSEVA/PDS pathology. Our findings can confirm the important role of SLC26A4 mutations in determining the phenotype of isolated EVA/PDS, both for the type/degree of the malformation, the hearing impairment and the association with thyroid dysfunction.

## Introduction

Approximately 20% of congenital hearing loss (SNHL) is associated with inner ear malformations (IEM) [1, 2]. The enlarged vestibular aqueduct (EVA) is reported to be the most common IEM associated with hearing loss in children, often bilaterally [3–5]. The IEM represent, thus, the third cause of hearing loss in the paediatric population [5]. IEM and, in particular EVA, can be an isolated disease (nonsyndromic) or represent part of a syndromic manifestation associated with specific genetic mutations [3, 6–8].

**Fig. 1:**
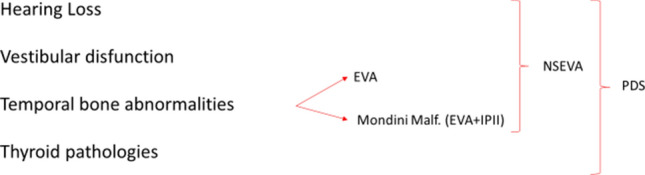
Description of the different features of non-syndromic enlarged vestibular aqueduct (NSEVA) and Pendred Syndrome (PDS); *EVA* enlarged vestibular aqueduct, *IP-II* incomplete partition type II

When referring to EVA, therefore, we should differentiate between nonsyndromic enlarged vestibular aqueduct (NSEVA or DFNB4) and Pendred Syndrome (PDS). Both conditions comprise a wide spectrum of hearing loss, vestibular disfunctions and temporal bone abnormalities, but thyroid pathologies are also present in PDS (Fig. 1). For these reasons, NSEVA and PDS should be considered part of a disease continuum [9, 10]. Furthermore, an EVA can be associated with other syndromic conditions, such as distal renal tubular acidosis (dRTA), branchio-oto-renal syndrome (BOR) or Waandenburg syndrome [3, 11, 12].

NSEVA/PDS is described as being associated with pathogenic sequence variants of Pendrin’s Gene (SLC26A4) in about half of the cases, either as homozygous or compound heterozygous mutations [13]. Even if most of the patients with NSEVA/PDS are reported as compound heterozygous, subjects with one or no pathogenic mutations of SLC24A4 have been reported [14, 15]. Mutations in FoXi1 or KCNJ10 genes, both associated or not with SLC26A4 mutations have been reported in a minority of patients (about 1% each) [16].

Additionally, the enlargement of the vestibular aqueduct can be associated with cochlear and modiolar defects, defining the Mondini Malformation: the association of EVA, enlarged vestibule and cochlear incomplete partition type 2 [2]

The aim of our study is to analyse the clinical and audiological features of a monocentric cohort of Caucasian patients with NSEVA/PDS; their genetic assessment and morphological inner ear features are also described in detail. Furthermore, a review of the recent literature is provided, and our results discussed in comparison with those reported in the literature.

## Materials and methods

We retrospectively reviewed the audiologic and personal anamnestic data of 66 patients with NSEVA/PDS followed by our audiology service.

The audiologic data were extracted from the last audiologic evaluation of each patient. In every audiologic assessment otoscopy, pure-tone audiometry (PTA) for air and bone threshold, speech audiometry, tympanometry and stapedial reflex examination study was ruled out. For patients under the age of six, a behavioural hearing testing and auditory brainstem responses (ABR), both for air and bone conduction thresholds, were obtained. Furthermore, for the aided patient [both with cochlear implant (CI) and hearing aids (HA)], a free field audiometry was performed with and without the device on.

Audiometry measures were carried out using the Interacoustics Clinical Audiometer AC40. When measuring the air conduction hearing threshold, we assigned a value of 125 dB to any frequency threshold over the maximum output limit of the audiometer (105 dB for 0.25 kHz and 125 dB for 0.5 and 1 kHz, 120 dB for 2 kHz). Any vibrotactile sensation was excluded.

All the patients over 16 years old underwent a vestibular assessment with vestibular caloric testing. Responses were recorded through an infrared GN Otometrics eye-tracking system.

All the enrolled patients underwent MRI and TC study for the evaluation of the petrous bone. We refer to isolated EVA when the anterior–posterior diameter of the vestibular aqueduct is larger than 1.5 mm, according to Valvassori and Clemis criteria [17]; we refer to Mondini Malformation as the association of EVA, enlarged vestibule and cochlear dysplasia with cystic apex (known as incomplete partition type II (IP-II) [2, 18].

All the patients with NSEVA/PDS who gave their consent to the procedure underwent a genetic research of SLC26A4 gene mutations with PCR. After extracting DNA from whole blood, mutation screening was completed by single-stranded conformational polymorphism and direct sequencing of the *SLC26A4* coding region. The patients who underwent the procedure were divided into 4 groups: we refer to M0 for patients without SLC26A4 mutation, M1 for patients with heterozygous mutation, M2 for patients with compound heterozygous mutations and M3 for patients with homozygous mutation.

Associations between SCL26A4 mutations groups (M0, M1, M2, M3) and continuous variables were determined using the ANOVA procedure and the Kruskal–Wallis test for nonparametric data. The t-test was used for comparing the means of thresholds in between mutations groups. Associations between SCL26A4 mutations groups and categorical variables (type of mutations) were determined using the Spearman’s rank correlation procedure. The significant independent variables, then, were analysed together by a multivariate model based on multiple linear regression. *P* values < 0.05 were considered as statistically significant. All analyses were performed using SPSS v.26 technology.

## Results

Of the 66 patients (132 ears) presenting NSEVA/PDS followed by our centre, 26 were males (39%) and 40 were females (61%) with a mean age of 26.2 years (1–69 years).

Thirty-nine patients presented a NSEVA (59%), whereas 18 presented PDS (27%); 9 patients (13%) presented other syndromic conditions: 5 cases of dRTA, 3 mild cognitive impairment and 1 case of chromosomic abnormalities.

An isolated EVA was reported in 47 patients (71%), unilaterally in 9 cases (14%, 5 left, 4 right); bilaterally in 38 cases (57%); meanwhile, a Mondini Malformation was found in 19 patients (29%), unilaterally in 1 case (1,5%), bilaterally in 18 cases (27%).

All the reported patients presented some grade of hearing loss, unilaterally in 10 cases (15%), bilaterally in 56 cases (85%). Considering each ear singularly, 10 ears (8%) had normal hearing, 9 ears (7%) a mild hearing loss, 21 ears (16%) a medium hearing loss, 15 ears (11%) a severe hearing impairment, and 77 ears (58%) a profound hearing loss. Twenty-one patients presented a mixed hearing loss (32%), with a mean air bone gap of 15.5 dB. No patients presented a pure conductive hearing loss.

Three of the 10 patients with unilateral malformation presented a bilateral hearing impairment. In 2 of these cases, the malformed ear presented a worse threshold, while in another case, the worse hearing threshold was reported in the anatomically normal ear. Moreover, 3 of the 10 patients with unilateral hearing impairment presented a bilateral malformation.

The mean age at hearing loss onset was 3.9 years (from congenital to 20 years). In 23 cases (35%), the hearing loss was congenital. Except for 7 congenital profound hearing-impaired patients (11%), all the other cases (89%) experienced a progression of hearing loss over time, often stepwise or trigger onset.

Thirty-two patients in the reported cohort (48%) used hearing aids for hearing rehabilitation, 21 patients (32%) had undergone a unilateral cochlear implantation, and 3 patients (4%) had been bilaterally implanted. The 10 patients that presented a single normal ear (15%) were unaided.

A vestibular impairment was reported in 34 of the 44 tested patients (77%), of whom 6 (17%) presented a bilateral vestibular areflexia at caloric vestibular stimulation. Otherwise, 10 patients (23%) did not manifest any vestibular disfunction at caloric stimulation.

Fifty-seven of the followed patients (86.3%, 114 ears) had been submitted to genetic research of SLC26A4 mutations. 24 patients (42.1%) had been found no mutations of Pendrin gene (M0); in 10 cases (17.5%), we found a heterozygous mutation (M1), in 14 patients (24.5%), a compound heterozygous mutation (M2) and in 9 patients (15.7%), a homozygous mutation (M3). In nine patients (13.6%), it was not possible to perform the genetic test, as the patients did not give their consent.

At univariate analysis, we found a significative association between SLC26A4 mutations and the presence of PDS rather than NSEVA (Spearman’s coefficient = 0.269 *p* < 0.019). Indeed, PDS was more likely to be present in the M3 or M2 groups than in the M1 and M0 groups.

Furthermore, SLC26A4 mutations were significantly associated with the presence of the different malformations associated with NSEVA/PDS (Spearman’s coefficient = 0.417 *p* < 0.001). In fact, Mondini Malformation was more likely to be present in the M3 group, followed by M2, M1 and then the M0 group. Isolated EVA, on the other hand, was significantly more frequent in patients of the M0 group, then M1, M2 and, finally, M3 group of patients.

**Fig. 2:**
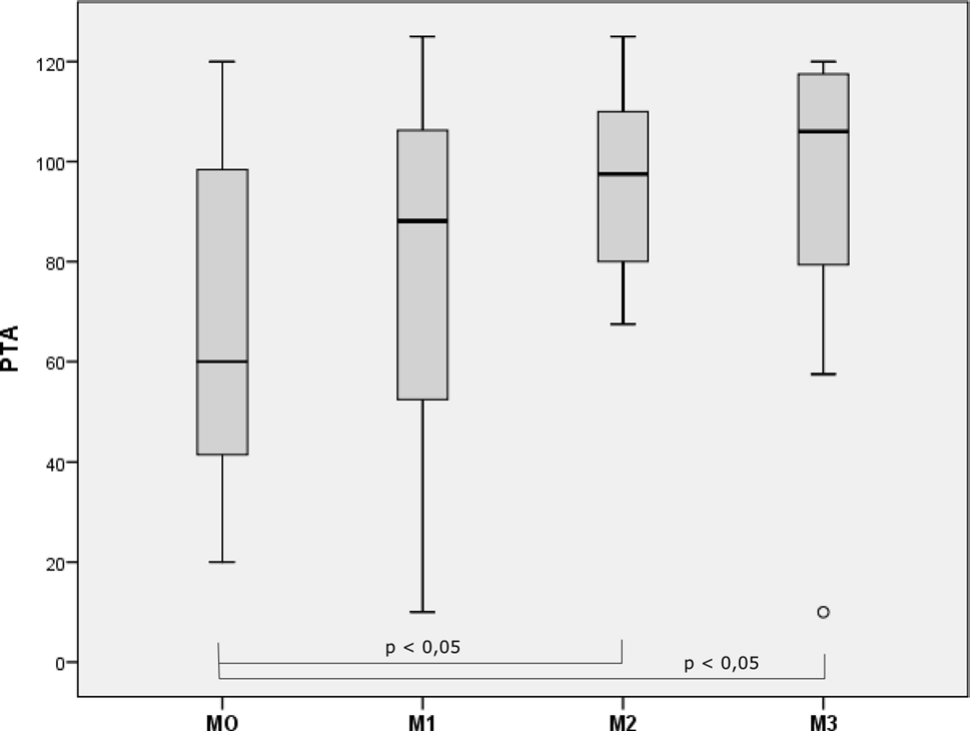
Whisker box plots of pure tone audiometry (PTA) in M0, M1, M2 and M3 group of patients; difference between the groups is statistically significative (*p* = 0.017); M0 = patients without SLC26A4 mutations, M1 = patients with a single heterozygous mutation of SLC26A4, M2 = patients with compound heterozygous mutation of SLC26A4, M3 = patients with homozygous mutation of SLC26A4

Moreover, we found that the SLC26A4 mutations were significantly correlated with the PTA of each patient’s ear (Spearman’s coefficient = 0.363 *p* = 0.001). Indeed, the M3 patients presented the worse thresholds, followed by the M2 cohort, then the M1 and M0 groups. At Kruskal–Wallis testing, the difference between groups was statistically significant (*p* = 0.017). No significant differences in hearing thresholds were found between the M0 and M1 groups (*p* = 0.39), M1 and M2 groups (*p* = 0.076), M1 and M3 groups (*p* = 0.3) or M2 and M3 groups (*p* = 0.733) at Student t-testing, but significant differences of thresholds were found between the M0 and M2 groups (*p* = 0.003) and M0 and M3 groups (*p* = 0.043) (Fig. 2).

Moreover, we found that the expression of PDS significantly correlated with the expressions of worse PTA (Spearman’s coefficient = 0.265 *p* = 0.002) and patients with PDS showed significantly worse PTA than patients with NSEVA (*p* = 0.001). Furthermore, also the expression of inner ear malformation was found to be correlated with PTA (Spearman’s coefficient = 0.233 *p* = 0.007). Patients with anatomically normal ears presented better PTA than ears associated with Mondini Malformation or isolated EVA. Even if a significantly worse PTA was found in malformed ears (both with Mondini Malformation and isolated EVA) rather than anatomically normal ears (*p* < 0.001), no statistically significant differences were found in PTA between patients with Mondini Malformation and isolated EVA (*p* = 0.916).

No significant correlation was found between mutations and age at hearing loss onset (Spearman’s coefficient = − 0.286 *p* < 0.113) or mutations and progression of hearing loss (Spearman’s coefficient = − 0.045 *p* < 0.791).

At multivariate analysis, the PTA in our cohort was still significantly related with the expression of PDS (RC = 3,628 *p* = 0.001) and with the presence of inner ear malformations (RC = 2.709 *p* = 0.008). On the other hand, the association between the mutations group and PTA lost its significance (RC = 1.152 *p* = 0.253).

## Discussion

In this paper, we provided a detailed description of clinical, audiological, radiological and genetic features of our cohort of 66 patients with EVA. NSEVA/PDS is a disease continuum underlying many cases of hearing loss, vestibular disfunction and IEM [3, 9, 10]. It is reported as the third cause of congenital hearing loss [16].

The main theory underlying the relationship between the enlargement of the vestibular aqueduct and the various aspects of hearing loss (severity, onset and type) is based on the impact that inner ear fluids pressure determine on inner ear structures [19, 20]. EVA, indeed, may be associated with a hydroelectrolytic imbalance of the inner ear fluids and with an increased inner ear pressure; so the hydrops of all the membranous labyrinth can lead to the destruction of cochlear and vestibular membranous structures and, finally, to hearing loss and vestibular impairment [20]. The high inner ear fluid pressure, furthermore, can limit the stapes movement causing the conductive component of the hearing loss [3]. Another mechanism that may explain the conductive component of the hearing loss is the third window effect induced by EVA, first described by Merchant in 2007 [21]. An EVA, indeed, produces a large communication between the bony vestibule and the cranial cavity, resulting in an air–bone gap, similar to that observed in superior semi-circular canal dehiscence [21].

A wide spectrum of hearing loss is associated with NSEVA/PDS, and most of the authors reported a bilateral severe-to-profound SNHL. Despite that, unilateral, mild or medium hearing impairments, and even normal hearing patients with NSEVA/PDS have been described in the literature [3, 4, 17, 22–24].

In our cohort, most of the patients presented a severe-to-profound hearing impairment [92 of the 132 ears (70%)]. Considering each ear singularly, 7% (9 ears) presented a mild hearing loss and 16% (21 ears) a medium hearing loss.

The hearing loss was bilateral in 56 cases (85%) and unilateral in 10 cases (14%). It is noticeable that even 3 out of 10 anatomically normal ears presented some degree of hearing loss. We can speculate that the molecular mechanism underlying the pathology may cause a hearing impairment even without a radiologically evident malformation of the inner ear.

Regarding the type of hearing loss, even if SNHL was reported as the most frequently associated with NSEVA/PDS, patients with mixed and pure conductive cases are also described in the literature [23] in a widely variable percentage: from 12 [25] to 80% [26]. In our cohort, 45 patients presented SNHL (68%) and 21 a mixed hearing loss (32%). No patients presented a pure conductive hearing loss.

A wide variability is also reported regarding hearing loss progression over time. Many patients present congenital forms of hearing loss, but some grade of progression or fluctuation of the hearing threshold is constantly described in the literature. The reported percentage of progressive hearing loss in patients with NSEVA/PDS varies from 17 [25] to 60% [27]. Even if a recent study reported that subjects with biallelic mutation of SLC26A4 showed earlier onset of hearing loss and faster progression, we could not find the same in our cohort. Trigger events are also stated as being related with the onset of hearing loss by many authors [3, 23]. The most frequently reported trigger is head trauma [28, 29], but also Valsalva manoeuvre [30], physical exercise [26], barotrauma or high fever [31] has been related to the onset of worsening hearing. The reported percentage of patients that experienced worsening hearing after trigger events goes from 3 [29] to 80% [32].

In the present study, we reported a mean age at hearing loss onset of 3.9 years, with 23 cases (35%) of congenital hearing impairment and a higher age at hearing loss onset of 20 years. Furthermore, 89% of patients experienced a progression of hearing loss over time, frequently stepwise or trigger related.

One of the most interesting and controversial aspects of NSEVA/PDS is the genetic aspect and the possibility of a correlation between SLC26A4 mutations and the phenotypic expression of the pathology.

In 1999, Usami et al. first demonstrated that mutations in the Pendrin gene were associated with the presence of both NSEVA and PDS [8]. Nowadays, it is widely demonstrated that NSEVA/PDS molecular diagnosis relies on the identification of a biallelic pathogenic mutation of *SLC26A4* (in homozygosis or compound heterozygosis), or a double heterozygosity for one pathogenic variant in *SLC26A4* and one pathogenic variant in either *FOXI1* or *KCNJ10 *[16]. Despite that, even cases of NSEVA/PDS with a single or no allele mutation of SLC26A4 have been reported in the literature [14, 15]. Phenotypic differences regarding ethnicity have also been reported. Tsukamoto et al. [33] and Park et al. [34] reported that in NSEVA/PDS Korean and Japanese patients more than 80% present two pathogenic mutations of SLC26A4, slightly more than 10% a single pathogenic mutation and slightly less than 10% no pathogenic mutation. In European and North American patients, 25% of cases are reported to present two pathogenic mutations of SLC26A4, another 25% a single pathogenic mutation and 50% no pathogenic variant of SLC26A4 [35, 36]. Our cohort of European Caucasian patients presented an incidence of mutation in line with those reported in the literature for European and North American populations, even if we found a slightly higher incidence of biallelic mutated patients and a lower incidence of patients with monoallelic mutations and no mutations: 40.2% exhibited two pathogenic mutations (24.5% as compound heterozygosis, 15.7% as homozygosis), 17.5% revealed a single pathogenic mutation and 42.1% showed no mutations.

Pendrin is a trans-membrane protein highly expressed in the thyroid and inner ear, and it is involved in the maintenance of inner ear fluid homeostasis [37, 38]. The lack of pendrin or the loss of its functionality during ontogenesis can determine an increase in inner ear fluid pressure due to an abnormal endolymphatichydro-electrolyte balance and osmotic pressure, leading to a dilated vestibular aqueduct [6, 12, 17, 39, 40]. Furthermore, the increasing fluid pressure through the EVA during ontogenesis can determine a dilatation of the vestibule and the cystic evolution of the apical part of the cochlea, as previously theorized by Sennaroglu et al., leading to the so-called Mondini malformation [40].

The hydroelectrolytic imbalance can also explain the association between EVA and other syndromes such as dRTA.

Previous studies correlated the genetic features in terms of number of mutated alleles of the SLC26A4 gene to the severity of the associated IEM, to the entity of the hearing impairment, to the tendency to progression over time of the hearing loss and to the expression of thyroid pathology.

It is speculated that a more severe impairment of pendrin functionality related to a biallelic mutation may determine a higher inner ear fluid pressure and so worse hearing thresholds and more severe malformations, namely EVA with wider diameters and Mondini Malformation.

In this regard, in 2019, Mey et al. demonstrated that individuals with two allele mutations of the Pendrin gene have a higher probability of having Mondini Malformation instead of isolated EVA [41, 42]. Moreover, the same authors reported that SLC26A4 biallelic mutations were correlated with larger endolymphatic sac size [41, 42]. Our findings confirmed the Mey et al. thesis. Patients with biallelic mutations (M2 and M3 groups), indeed, were more likely to present Mondini Malformation than isolated EVA.

Furthermore, King et al. in 2010, Rose et al. in 2017, and Mey et al. in 2019 demonstrated that patients with 2 mutated alleles showed worse thresholds and a tendency to progress faster than the patients with 1 or no mutated SLC26A4 alleles [22, 41–43]. Also, an earlier onset of hearing loss in patients with biallelic mutations was described by those authors. On the other hand, other authors have published studies that showed no correlations between SLC26A4 gene mutations and hearing loss features [24, 44]. Our findings partially confirm what has been previous reported in the literature. As a matter of fact, in our cohort, patients with biallelic mutations (M2 and, in particularly, M3 groups) presented the worse thresholds with a statistically significant difference between the groups (*p* = 0.017): no significant differences in hearing thresholds had been found between the M0 and M1 groups, M1 and M2 groups, M1 and M3 groups, or M2 and M3 groups, but significant differences of thresholds were found between the M0 and M2 groups and M0 and M3 groups.

Otherwise, no significant correlations were found between mutations and age at hearing loss onset or tendency to hearing loss progression.

Regarding the petrous bone malformations, we found a significant correlation between the SLC26A4 mutations group and the presence of Mondini Malformations rather than isolated EVA, with Mondini Malformation more likely to be present in the M3 group, followed by M2, M1 and then the M0 group and isolated EVA more frequent in patients of the M0 group, then M1, M2 and, finally, the M3 group of patients. In our sample, we did not find significant differences in the thresholds of patients with isolated EVA compared with those presenting a Mondini Malformation, similar to King et al. [20], who did not find any correlation between the degree of inner ear anomalies and the severity of hearing loss in NSEVA/PDS. On the other hand, anatomically normal ears presented a significantly better PTA than malformed ears.

Regarding the association between EVA and thyroid dysfunction, Azaiez et al. reported that biallelic mutations of SLC26A4 are also correlated with PDS expression [45]. Our findings confirm the findings of the study by Azaiez and colleagues, since we also reported that PDS was more likely to be present in the M3 or M2 groups than in the M1 and M0 groups with a significant association.

Finally, patients with PDS showed significantly worse PTA than patients with NSEVA.

From the literature data and from those herein reported, we can confirm the important role of SLC26A4 mutations in determining the phenotype of isolated EVA/PDS, both for the type/degree of the malformation, the hearing impairment and the association to thyroid dysfunction. A biallelic mutation of the SLC26A4 gene (especially if homozygous) can lead to a more severe pathology, with increasing ear homeostasis impairment and consisting of a more severe hearing loss, more severe IEM and a higher chance of presenting PDS rather than NSEVA.

## Conclusions

NSEVA/PDS is a disease continuum presenting a wide spectrum of hearing impairment, vestibular dysfunction, IEM, and thyroid pathologies. Specific genetic mutations of the SLC26A4 gene underlie this pathology.

Furthermore, as the findings of our study on European Caucasian subjects confirmed, patients that have biallelic mutations (especially if homozygous) express a more severe disease, with worse hearing thresholds and a higher tendency to present thyroid pathologies and Mondini Malformations rather than isolated EVA.

Given the relatively higher prevalence and the widely variable audiologic presentation, NSEVA/PDS must be investigated in all the congenital hearing loss, but also in progressive, late onset, stepwise forms. Even mixed or fluctuating hearing loss may constitute a sign of a NSEVA/PDS pathology.

In patients suspected to present NSEVA/PDS, a diagnostic assessment should include a complete audiological evaluation and vestibular testing to characterize the audio-vestibular features of each subject, a petrous bone radiologic assessment for the evaluation of malformative aspects, a thyroid functionality testing and the genetic study for defining SLC26A4 mutations.

Once the diagnosis is completed, therefore, it is important to set up accurate counselling sessions with the patients or their family, underlining the possibility that the hearing loss will progress and the importance of avoiding triggers like head trauma, Valsalva manoeuvre, and scuba diving.

Even if our findings confirmed what some previous studies have reported in the literature with a rather large cohort of European Caucasian patients, further research on larger cohorts of patients are needed to better understand the pathogenic mechanism underlying NSEVA/PDS.

## Data Availability

All the data are stored at our clinic’s archives.
